# Acute Myeloid Leukemia Complicated by Hyperleukocytosis and Leukostasis in the Emergency Department

**DOI:** 10.7759/cureus.15392

**Published:** 2021-06-02

**Authors:** Ryan T Ngo, Amanda L Webb McAdams, Anthony Furiato

**Affiliations:** 1 Emergency Medicine, Brandon Regional Hospital, Brandon, USA

**Keywords:** acute myeloid leukemia, hyperleukocytosis, leukostasis, hematologic malignancies, blast crisis

## Abstract

Acute myeloid leukemia (AML) and other hematologic malignancies can be complicated by hyperleukocytosis, which leads to an increased risk for other severe complications such as tumor lysis syndrome, disseminated intravascular coagulation (DIC), and leukostasis. In this report, we present a case of a 65-year-old female with newly diagnosed AML complicated by leukostasis. We briefly review the clinical significance as well as initial diagnostic and therapeutic considerations pertaining to hyperleukocytosis and its associated complications.

## Introduction

Leukostasis is a complication of hyperleukocytosis characterized by the presence of end-organ injury in patients with underlying hematologic malignancy. It is prevalent in 15-45% of patients presenting with acute myeloid leukemia (AML) and hyperleukocytosis, and it is associated with a one-week mortality rate of more than 20% [1]. There are no definitive criteria to guide its diagnosis, and these patients can deteriorate rapidly. In the right clinical setting, it is therefore important to maintain a high index of suspicion for its presence, and it is critical to have a good understanding of the initial steps of evaluation and management of hyperleukocytosis and its associated complications.

## Case presentation

**Figure 1:**
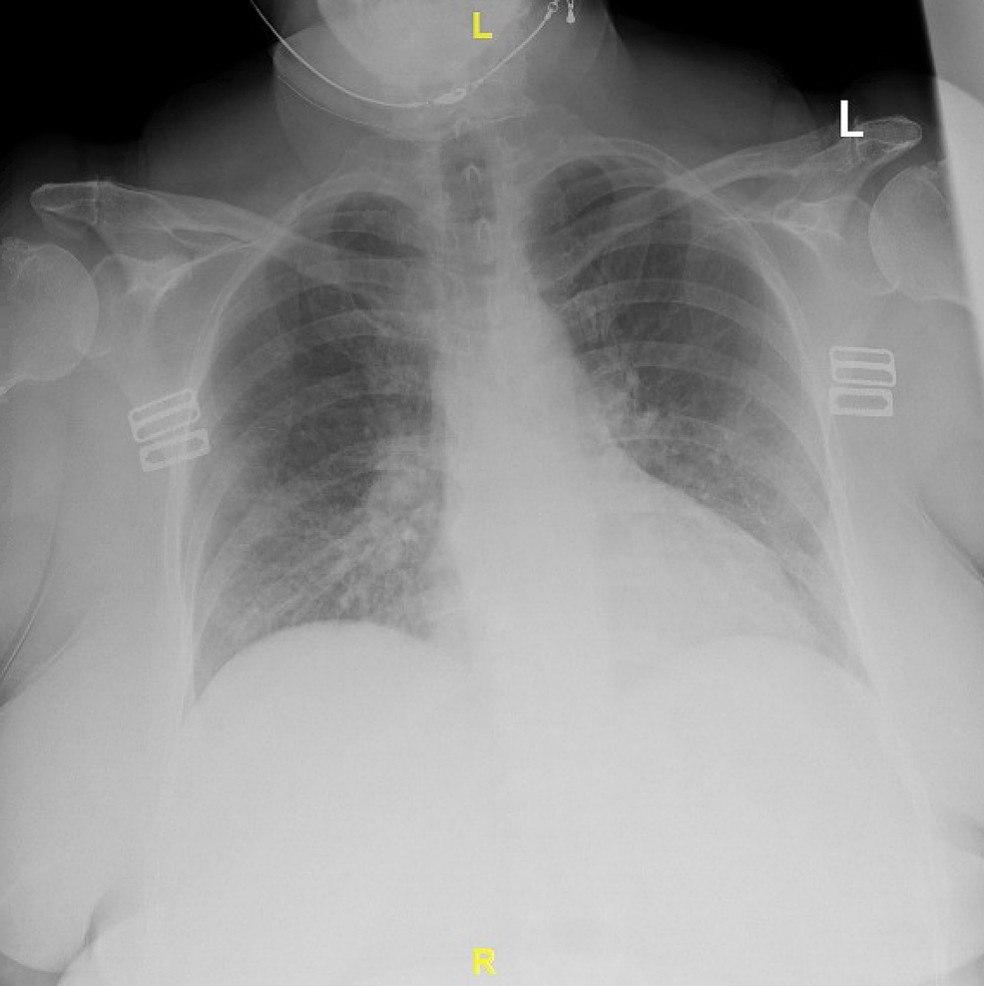
Chest X-ray The image shows mild pulmonary edema

A 65-year-old female with a history of smoking and breast cancer in remission following lumpectomy, chemotherapy, and radiation five years prior presented to the emergency department with complaints of fevers, exertional dyspnea, vomiting, diarrhea, and melena for three days. Her vital signs were significant for an oxygen saturation of 89% on room air but were otherwise normal. Physical examination revealed a morbidly obese, ill-appearing woman with tenderness to the epigastrium and hepatomegaly but was otherwise unremarkable. Laboratory findings were notable for hyperleukocytosis, macrocytic anemia, thrombocytopenia, acute renal failure, transaminitis, non-ST segment elevation myocardial infarction (NSTEMI), elevated brain natriuretic peptide (BNP), and lactic acidosis (Tables 1, 2). Chest X-ray showed mild pulmonary edema (Figure 1). CT of the abdomen and pelvis showed hepatomegaly. EKG was unremarkable except for a prolonged QTc interval.

**Table 1:** Complete blood count with differentials

Test	Result
White blood cell count	526.7 x 10^3^/uL
Hemoglobin	5.6 g/dL
Hematocrit	18.4%
Mean corpuscular volume (MCV)	115.0 FL
Platelet count	115 x 10^3^/uL
Immature granulocytes	4.8% (25.75 K/MM^3^)
Neutrophils	2.1% (11.7 K/MM^3^)
Lymphocytes	3.5% (18.7 K/MM^3^)
Monocytes	89.5% (480.3 K/MM^3^)
Eosinophils	0.0% (0.1 K/MM^3^)
Basophils	0.1% (0.3 K/MM^3^)

**Table 2:** Complete metabolic panel, cardiac enzymes, lactic acid, and procalcitonin levels BUN: blood urea nitrogen; GFR: glomerular filtration rate; AST: aspartate aminotransferase; ALT: alanine aminotransferase

Test	Result
Sodium	129 mmol/L
Potassium	3.8 mmol/L
Chloride	94 mmol/L
Carbon dioxide	20 mmol/L
Anion gap	15
BUN	63 mg/dL
Creatinine	7.95 mg/dL
GFR calculation	5 mL/min/1.73 m^2^
Glucose	179 mg/dL
Calcium	9.5 mg/dL
Total bilirubin	7.0 mg/dL
AST	113 Units/L
ALT	270 Units/L
Alkaline phosphatase	368 Units/L
Troponin I	0.099 ng/mL
Pro-B-type natriuretic peptide	20,574 pg/mL
Lactic acid	4.8 mmol/L
Procalcitonin	3.34 ng/mL

Per the hematologist's recommendation, the patient was transferred to a facility equipped with leukapheresis capabilities. Blood cultures ultimately showed no growth at five days. Subsequent evaluations including bone marrow aspiration and biopsy and cytogenetic studies were not available; however, flow cytometry showed increased blasts (83% of cells) with immunophenotypic features consistent with AML with monocytic maturation. Lymphoid mature B cells, plasma cells, T-cells, and NK cells appeared in normal proportions and were also immunophenotypically normal. Morphology and immunophenotypic analyses on peripheral blood along with the patient’s history were most consistent with AML with a World Health Organization (WHO) classification of therapy-related myeloid neoplasm and a French-American-British (FAB) classification of M5.

## Discussion

AML is the most common type of acute leukemia in adults [2]. As the bone marrow is replaced by poorly differentiated myeloid precursors, a consequent reduction in the number and function of other cellular lineages occurs, leading to variable degrees of functional and absolute cytopenia. This can manifest in patients as fatigue, bleeding, and infections, as seen in our patient who complained of melena [3]. Although this can easily be identified on routine laboratory analysis, recognition of other less common but more emergent complications is paramount for initiating appropriate workup and resuscitation.

Approximately 6-20% of patients with AML are found to have hyperleukocytosis, which is commonly defined as having >100,000 WBC/ul [4]. The presence of hyperleukocytosis carries an increased risk for the development of tumor lysis syndrome, disseminated intravascular coagulation (DIC), and leukostasis, which can occur in up to 30%, 30%, and 45% of patients, respectively [5].

Rapid cellular turnover and breakdown in tumor lysis syndrome can result in hyperuricemia, hyperkalemia, hypocalcemia, and hyperphosphatemia and can predispose to the development of acute renal failure [6]. Treatment includes early and aggressive hydration and the medications allopurinol as well as rasburicase for those with insufficient improvement [7]. Although our patient suffered from acute renal failure, there were no observed abnormalities in potassium or calcium levels.

DIC in patients with AML has been inconsistently associated with higher relative rates of thrombosis compared to bleeding [8,9]. Bleeding and thrombotic events have been found to be the most common cause of early mortality in these patients, and intracranial hemorrhage is found in a large proportion among them [5]. Evaluation includes measurement of coagulation profiles, including fibrinogen and fibrin degradation products. Therapy is generally aimed at reversing the coagulopathy through the transfusion of cryoprecipitate, platelets, fresh frozen plasma, and occasionally heparin depending on the underlying deficiencies, chronicity, and symptoms that predominate [8].

Leukostasis is characterized by the formation of microvascular white blood cell plugs that arise from hyperviscosity caused by increasing leukocyte volumes as well as increased leukocytic tissue infiltration due to altered expression of inflammatory mediators [10,11]. Although it most commonly manifests with pulmonary and central nervous system complaints, myocardial ischemia, renal injury, and bowel infarction have also been documented [1]. Its presence denotes a significant increase in early morbidity and mortality and constitutes a true hematologic emergency due to the possibility of rapid deterioration [5]. Definitive treatment requires leukemia-specific induction therapy; however, patient-specific factors and comorbid acute illnesses such as renal failure can preclude timely cytoreduction with intensive chemotherapy [12]. Alternative options include myelosuppression with hydroxyurea, non-intensive cytotoxic agents, and mechanical reduction with leukapheresis. Hydroxyurea can be effective in up to 75% of patients within four days and decreases in-hospital mortality rates [13,14]. Alternative non-intensive chemotherapeutic agents can likewise be limited by renal function or lack of expediency, though they may still represent viable options. Leukocytapheresis is effective at reducing leukocyte counts, but data on its effect on early mortality and associated complications is conflicting, and there is no indication that it improves overall survival or long-term outcomes [5,15-17]. Regardless, the American Society of Apheresis (ASFA) has recommended leukapheresis as a second-line therapy for patients with leukostasis based on moderate-quality evidence (category II, grade 2B recommendation) and considers leukapheresis to have a poorly defined role in asymptomatic hyperleukocytosis based on low-quality evidence (category III, grade 2C recommendation) [18].

The patient in our case presented with leukostasis manifesting as acute hypoxemic respiratory failure but had a notably severe degree of hyperleukocytosis relative to some other cases. Her symptoms and vital sign abnormalities were relatively mild given her underlying laboratory findings and multi-organ dysfunction. Her concurrent renal failure highlights the importance of coordinating the care of such patients with a hematologist as alternative cytoreductive efforts such as leukapheresis must be considered in cases of any delay in initiating definitive inductive therapy.

## Conclusions

AML can manifest in patients with symptoms related to the underlying pancytopenia or other associated complications. Hyperleukocytosis, when present, should prompt a high suspicion for the presence of tumor lysis syndrome, DIC, and leukostasis. An awareness of these associations is paramount for initiating appropriate workup at the time of presentation, which may often be in the emergency department. This may ultimately lead to more expeditious and appropriate care, aided by coordination with the hematologist for further management.
